# The immunomodulatory effects of long-term supplementation with *Lactobacillus casei* Shirota depend on ovalbumin presentation in BALB/c mice

**DOI:** 10.1038/s41598-021-98791-2

**Published:** 2021-09-30

**Authors:** Li-Han Chen, Chun-Hsu Pan, Shih-Yi Huang, Ching-Hung Chan, Hui-Yu Huang

**Affiliations:** 1 Institute of Fisheries Science, National Taiwan University, Taipei, 10617 Taiwan; 2 Department of Life Science, National Taiwan University, Taipei, 10617 Taiwan; 3 School of Pharmacy, Taipei Medical University, Taipei, 11031 Taiwan; 4 Graduate Institute of Metabolism and Obesity Sciences, Taipei Medical University, Taipei, 11031 Taiwan; 5 Department of Chemical Engineering and Biotechnology, Tatung University, 10452 Taipei, Taiwan

**Keywords:** Nutrition, Applied microbiology

## Abstract

Immunomodulation is an ability of several particular probiotics. However, it still remains unclear whether the immunomodulatory effects of specific probiotics vary for different antigen presentation models with the same antigen. To investigate this matter, six groups of BALB/c mice (*n* = 10) were exposed to one of two antigen presentation models: ovalbumin (OVA) by injection alone, or injection plus intranasal administration. Moreover, the mice were fed distilled water or *Lactobacillus casei* Shirota fermented beverage (LcSFB) at low (2.5 × 10^9^ CFU/kg body weight) or high doses (5 × 10^9^ CFU/kg body weight) by gavage for 8 weeks. LcSFB enhanced the proliferation of splenocytes, production of OVA-specific immunoglobulin (Ig)-G and IgA, and the ratio of T-helper (Th)-2/Th1 cytokines in mice injected with OVA. Conversely, in the mice treated with OVA by injection plus intranasal administration, LcSFB attenuated the immune responses against OVA by reducing the proliferation of splenocytes, levels of OVA-specific IgE, IgG, and IgM, and ratio of Th2/Th1 cytokines. Moreover, LcSFB increased the percentage of regulatory T cells in the injection plus intranasal administration group. Taken together, this work indicates the immunoregulatory effects of LcSFB depend on how the antigen is presented. Therefore, the use of probiotics to boost the immune system must be carefully considered.

## Introduction

Probiotics are defined by the World Health Organization as ‘live micro-organisms’ that are used to ferment food and can confer health benefits to their hosts^1^. The panel convened in October 2013 by the International Scientific Association for Probiotics and Prebiotics (ISAPP) further recommended the term probiotic be used only on products that deliver live microorganisms with a suitable viable count of well-defined strains with a reasonable expectation of delivering benefits for the wellbeing of the host^2^. Over time, an increasing number of bacteria have been used as probiotics and shown to improve several disorders^3–5^, particularly conditions related to imbalanced immunity^6–8^. Although probiotics have been shown to confer immunoregulatory effects in previous studies, the effects of probiotics are strain-dependent^9,10^. For example, *Lactobacillus paracasei* DC412 and *L. acidophilus* NCFB 1748 enhance immune responses against exogenous antigens^11^. However, *L. rhamnosus* HN001 and *L. fermentum* VR1-003PCC attenuate immune responses to prevent allergies^12^.

Interestingly, Hori et al*.* (2002) showed that *Lactobacillus casei* Shirota (LcS) boosted immunity against the influenza virus^13^. However, another study reported that LcS exhibited immune-suppressing effects in subjects with asthma^14^. These results suggest that supplementation with the same probiotic may lead to different effects on immunity, and using probiotics for immune regulation may lead to undesired effects. Therefore, it is necessary to understand why probiotics result in different immune-related consequences.

One of the differences between the two LcS studies noted above is the antigen presentation model. Although the antigen was presented via injection in both studies, intranasal administration of the antigen was only used in the latter study. Intranasal administration allows antigens to settle on the mucosa, whereas injections send antigens directly into the circulatory system. Compared with the circulatory immune system, the mucosal immune system must tolerate non-pathogen antigens in microbiota, food, and airborne species, as the mucosa is the region of the body that comes into contact with the largest numbers of environmental antigens^15^. Therefore, the mucosal immune system employs a variety of mechanisms to prevent diseases related to immune hypersensitivity. Accordingly, it is easier to trigger immune tolerance via mucosal surfaces than via the circulatory system^16^. However, no studies have employed two different antigen presentation models in the same model to assess whether the antigen presentation model can influence the immune regulatory effect of a single probiotic.

In this study, we attempted to understand the effect of LcS-regulated immunity by using different antigen presenting models for the same antigen. Common antigen presentation models that simulate injective vaccination and allergic asthma were selected, as these models are well established antigen presentation methods. The antigen is presented to the host via intradermal injection alone in the injective vaccination model, but by intradermal injection combined with intranasal administration in the allergic asthma model. Ovalbumin (OVA) was chosen as a model antigen due to its well-established antigenic properties and capacity to induce host immunity. Then, the proliferation of splenocytes, levels of immunoglobulin (Ig) and cytokines, and percentages of regulatory T (Treg) and T helper (Th) cells were determined to evaluate the OVA-induced immune responses and evaluate the regulation of these immune responses by LcS in different antigen presentation models.

## Results

### LcS fermented beverage (LcSFB) increases the proliferation of splenocytes from OVA-injected mice

**Figure 1:**
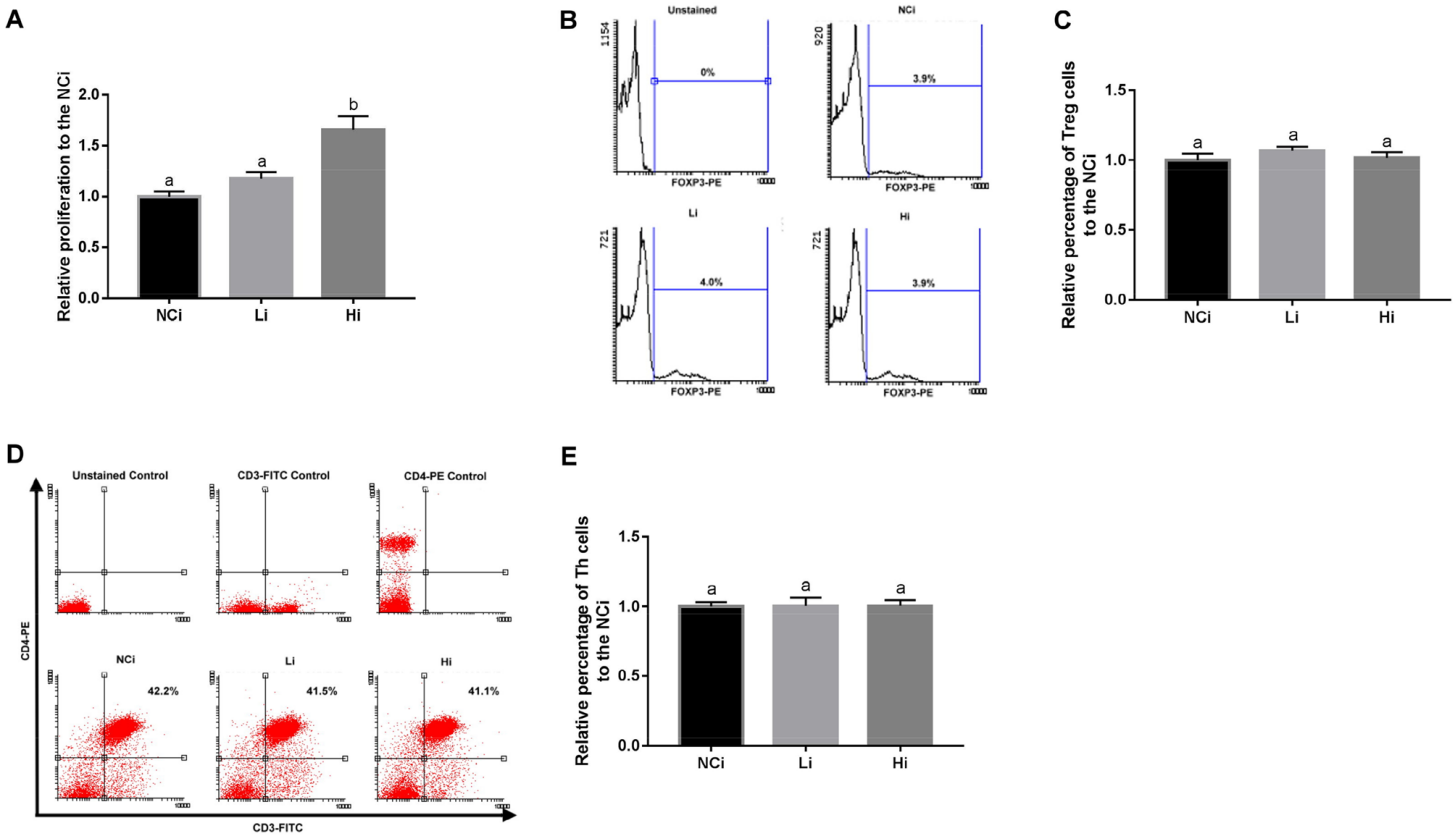
Effects of LcSFB on splenocytes from OVA-injected (s.c.) mice ex vivo. (**A**) Splenocyte proliferation. (**B**) Representative experiments evaluating the effect of LcSFB on Treg cells. (**C**) Relative percentages of Treg cells in splenocytes. (**D**) Representative experiments evaluating the effect of LcSFB on T helper cells (**E**) Relative percentages of T helper cells in splenocytes. *NCi* mice gavaged with saline, negative control, *Li* mice gavaged with low-dose LcSFB (2.5 × 10^9^ CFU/kg BW), *Hi* mice gavaged with high-dose LcSFB (5 × 10^9^ CFU/kg BW). Different superscript letters (a, b) indicate significant differences at *p* < 0.05; one-way ANOVA with Tukey HSD post-hoc tests (*n* = 10).

The effect of LcSFB on the OVA-induced immune response was first evaluated based on the proliferation of splenocytes and proportion of immune cells in splenocytes. The splenocytes of OVA-injected mice were stimulated with OVA ex vivo and proliferation was evaluated using the MTT assay. The results revealed that LcSFB increased the proliferation of splenocytes (Fig. 1A). However, LcSFB did not influence the percentages of Treg (Fig. 1B,C) or Th (Fig. 1D,E) cells.

### OVA-specific IgA and IgG are boosted by LcSFB in OVA-injected mice

**Figure 2:**
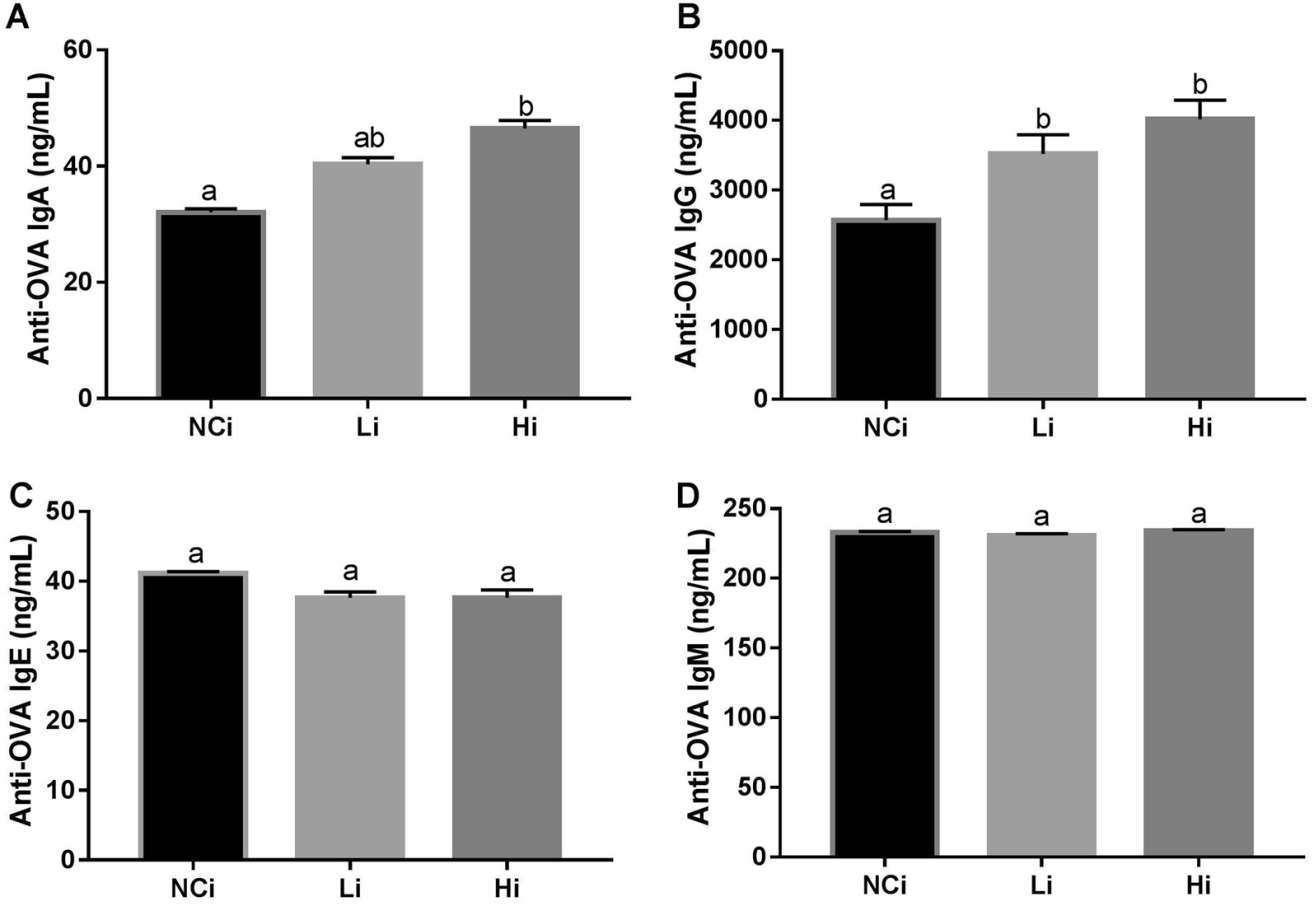
Concentrations of OVA-specific immunoglobulins in the serum of OVA-injected (s.c.) mice. Levels of the OVA-specific immunoglobulins (**A**) IgA, (**B**) IgG, (**C**) IgE, and (**D**) IgM in the serum. *NCi* mice gavaged with saline, negative control, *Li* mice gavaged with low-dose LcSFB, *Hi* mice gavaged with high-dose LcSFB. Different superscript letters (a, b) indicate significant differences at *p* < 0.05; one-way ANOVA with Tukey HSD post-hoc tests (*n* = 10).

As splenocytes contain a varied population of immune cells, the levels of immunoglobulin (Ig) in serum were also measured after OVA injection to evaluate the effect of LcSFB on the immune response. The concentrations of OVA-specific IgA (Fig. 2A) and IgG (Fig. 2B) were higher in OVA-injected mice that received high-dose LcSFB treatment than in the control group (NCi). However, the IgE (Fig. 2C) and IgM (Fig. 2D) levels of the Li and Hi groups did not differ to those of the NCi group.

### LcSFB enhances the levels of Th2-related cytokines in OVA-injected mice

**Figure 3:**
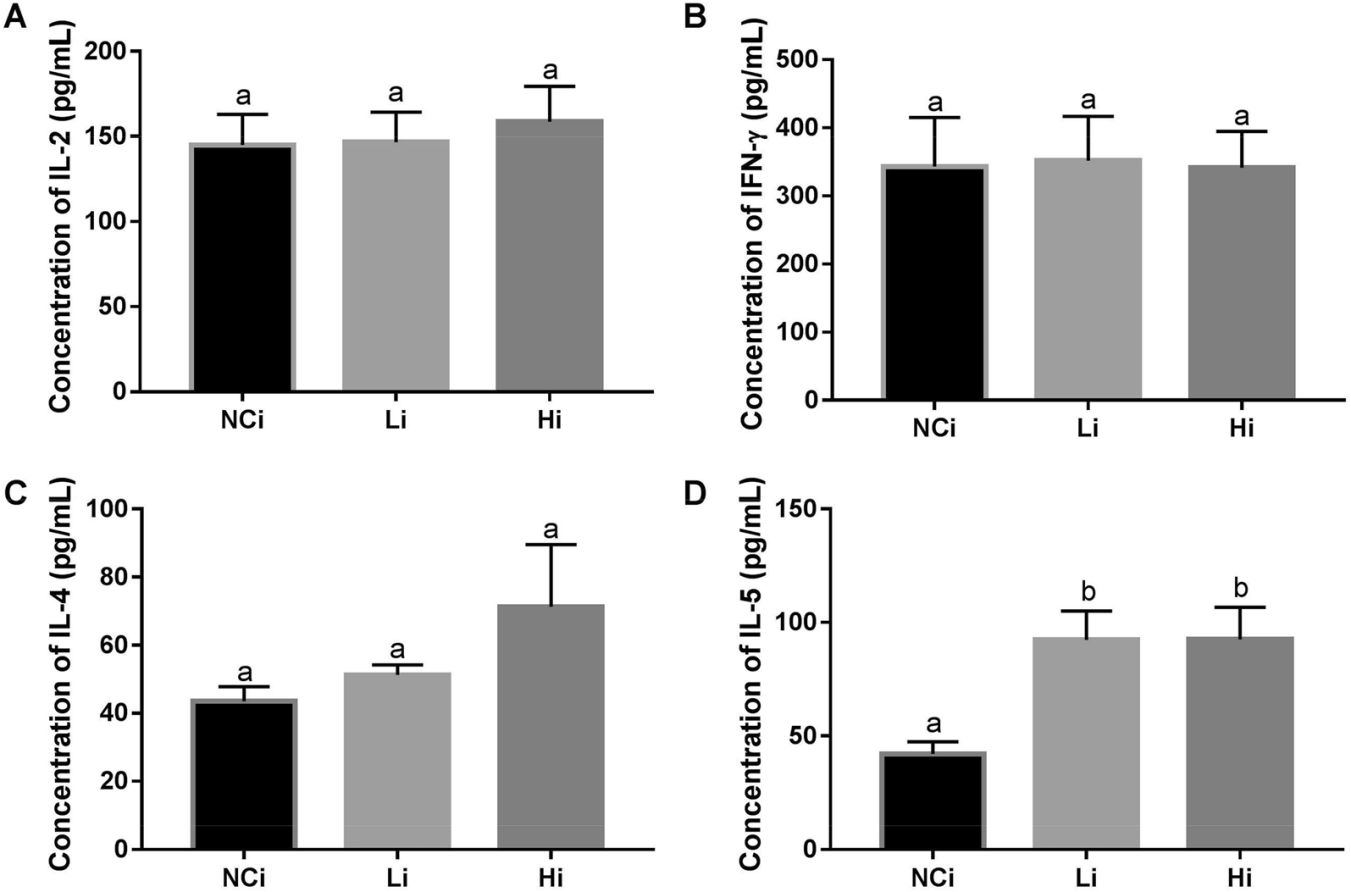
Cytokine profiles of splenocytes from OVA-injected (s.c.) mice ex vivo. Levels of the cytokines (**A**) IL-2, (**B**) IFN-γ, (**C**) IL-4, and (**D**) IL-5 in the supernatant. *NCi* mice gavaged with saline, negative control, *Li* mice gavaged with low-dose LcSFB, *Hi* mice gavaged with high-dose LcSFB. Different superscript letters (a, b) indicate significant differences at *p* < 0.05; one-way ANOVA with Tukey HSD post-hoc tests (*n* = 10).

Th1- and Th2-related cytokines were also measured to assess the immune response to OVA. After splenocytes from OVA-injected mice were stimulated with OVA ex vivo, the levels of Th1-related cytokines, interleukin (IL)-2 (Fig. 3A), and IFN-γ (Fig. 3B) were not significantly different among the NCi, Li, and Hi groups. The Th2-related cytokine IL-4 was not significantly increased in the Li and Hi groups (Fig. 3C); however, the levels of IL-5, another Th2-related cytokine (Fig. 3D), were significantly higher in the Li and Hi groups than the NCi group. Thus, LcSFB promoted an immune response in Th2 cells, but not Th1cells.

### LcSFB attenuates OVA-induced proliferation of splenocytes from mice injected with OVA and administered OVA intranasally

**Figure 4:**
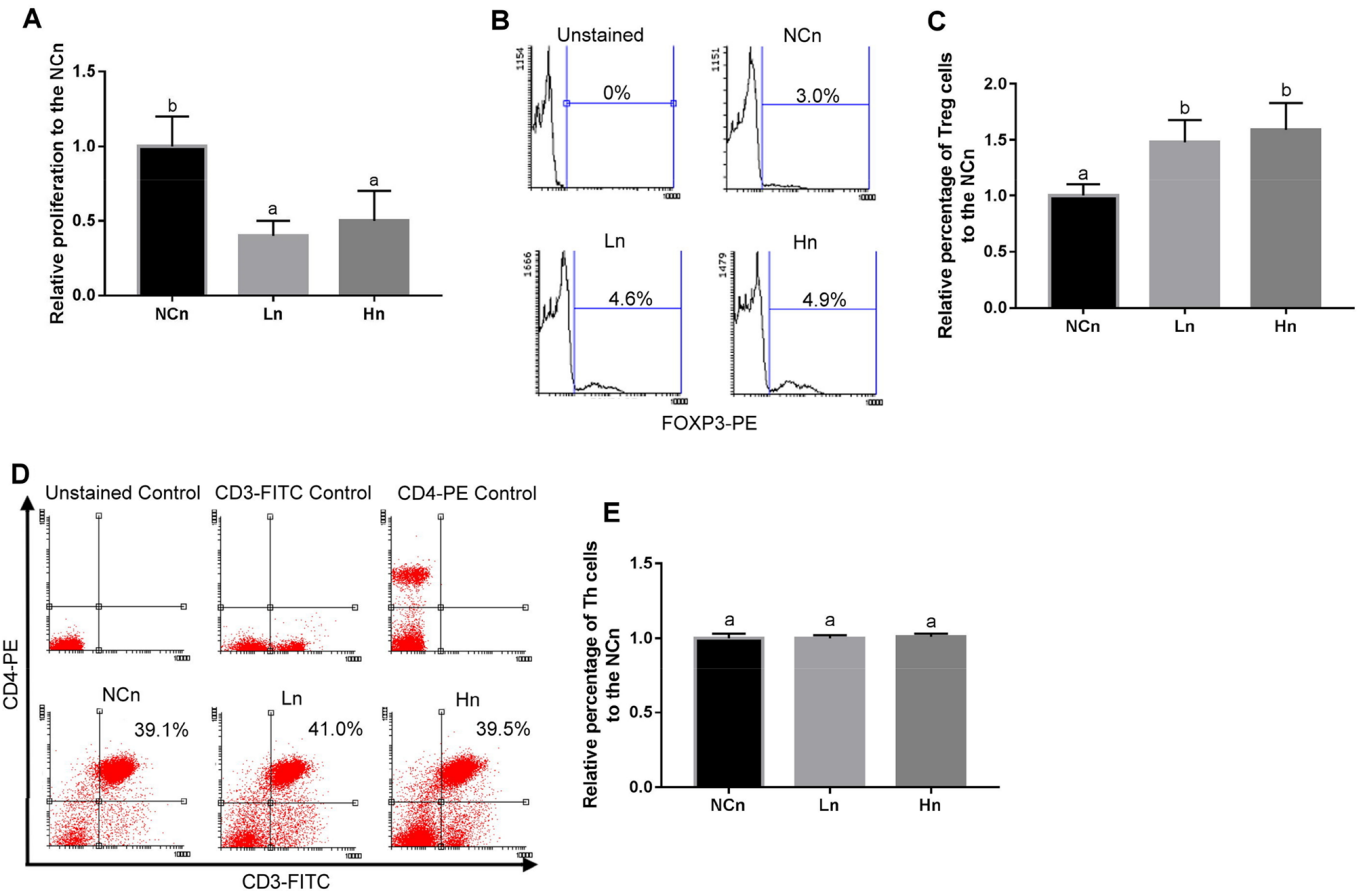
Effects of LcSFB on splenocytes from OVA-injected (s.c.) mice with additional intranasal administration ex vivo. (**A**) Proliferation of splenocytes. (**B**) Representative experiments evaluating the effect of LcSFB on Treg cells. (**C**) Relative percentage of Treg cells in the splenocytes. (**D**) Representative experiments evaluating the effect of LcSFB on T helper cells. (**E**) Relative percentage of T helper cells in splenocytes. *NCn* mice gavaged with saline, negative control, *Ln* mice gavaged with low-dose LcSFB, *Hn* mice gavaged with high-dose LcSFB. Different superscript letters (a, b) indicate significant differences at *p* < 0.05; one-way ANOVA with Tukey HSD post-hoc tests (*n* = 10).

To investigate whether different methods of antigen administration influenced the effects of LcSFB on the immune response, 12 OVA-injected mice were also treated with OVA via the nasal route for 3 days in the fourth and eighth weeks. In contrast to the immune-promoting effects of LcSFB observed in the OVA-injected mice (Fig. 1A), LcSFB decreased OVA-induced proliferation of splenocytes in OVA-injected mice that were also administered OVA via the nasal cavity (Fig. 4A).

### Treg cells are induced by LcSFB in mice injected with OVA and administered OVA intranasally

As our results indicated that LcSFB suppressed the proliferation of splenocytes in the OVA-injected mice that were also treated with OVA nasally, we next attempted to understand the effect of LcSFB on the immune suppressive cells, Tregs. LcSFB increased the percentage of Treg cells in splenocytes in OVA-injected mice that were also administered OVA intranasally (Fig. 4B,C). Moreover, LcSFB had no effect on the percentage of Th cells (Fig. 4D,E).

### Nasal OVA treatment alters the effects of LcSFB on immunoglobulin production in OVA-injected mice

**Figure 5:**
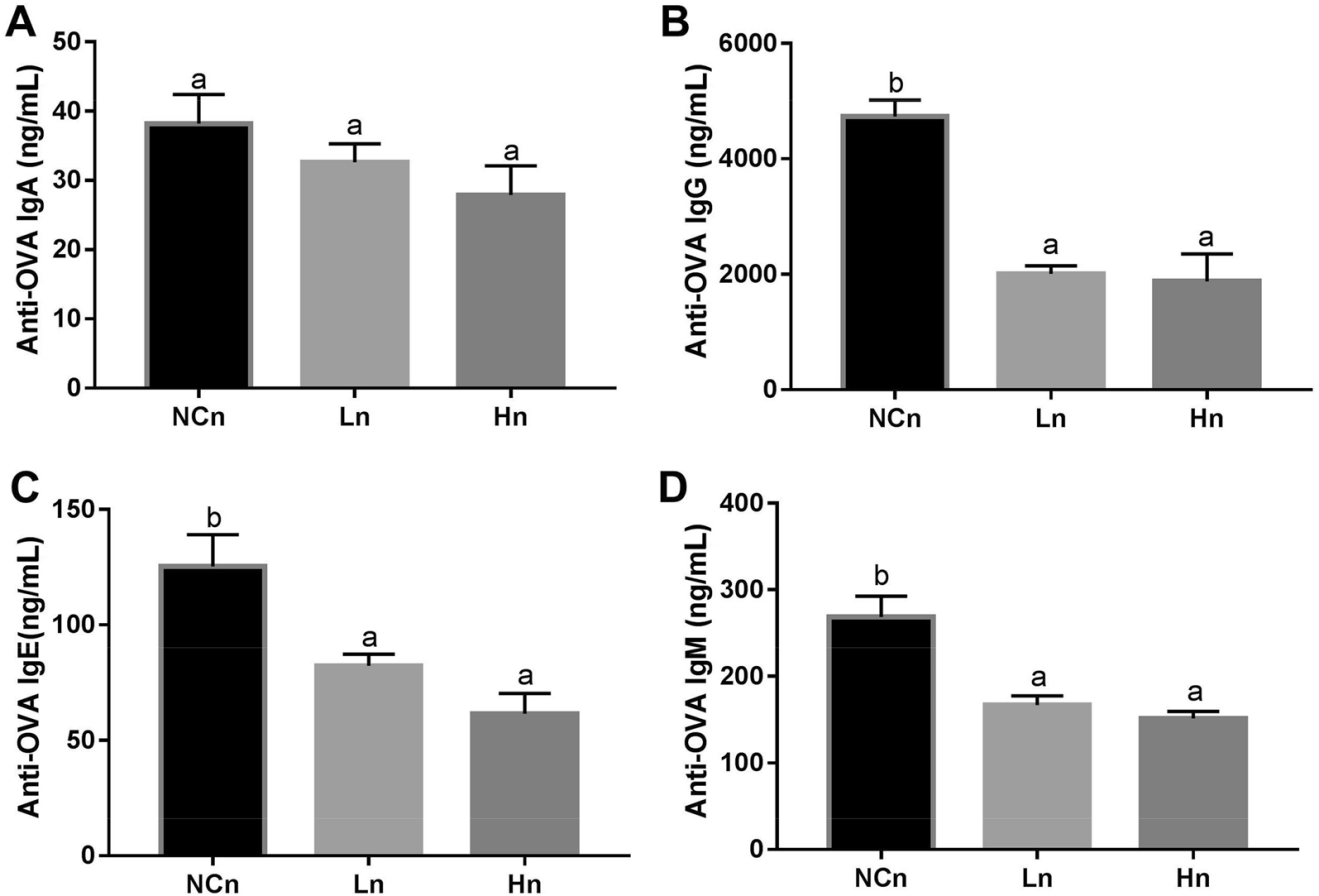
Concentrations of OVA-specific immunoglobulins in the serum of OVA-injected (s.c.) mice with additional intranasal administration. Levels of the OVA-specific immunoglobulins (**A**) IgA, (**B**) IgG, (**C**) IgE, and (**D**) IgM in serum. *NCn* mice gavaged with saline, negative control, *Ln* mice gavaged with low-dose LcSFB, *Hn* mice gavaged with high-dose LcSFB. Different superscript letters (a, b) indicate significant differences at *p* < 0.05; one-way ANOVA with Tukey HSD post-hoc tests (*n* = 10).

Intranasal OVA treatment enhanced the levels of anti-OVA IgE and IgG from 41.2 ± 0.2 ng/mL (Fig. 2C) to 125.3 ± 13.8 ng/mL (Fig. 5C) and from 2.57 ± 0.23 (Fig. 2B) to 4.73 ± 0.28 µg/mL (Fig. 5B), respectively, in OVA-injected mice fed saline. Furthermore, LcSFB significantly attenuated the effect of nasal OVA treatment, as lower levels of IgE and IgG were observed in the Ln and Hn groups than the NCn group (Fig. 5C,B). IgM levels were also significantly reduced by LcSFB (Fig. 5D). There was no significant difference in the IgA level between any group (Fig. 5A). Thus, LcSFB had no effect on IgA levels.

### Intranasal OVA treatment affects the regulatory effects of LcSFB on Th1 and Th2 in OVA-injected mice

**Figure 6:**
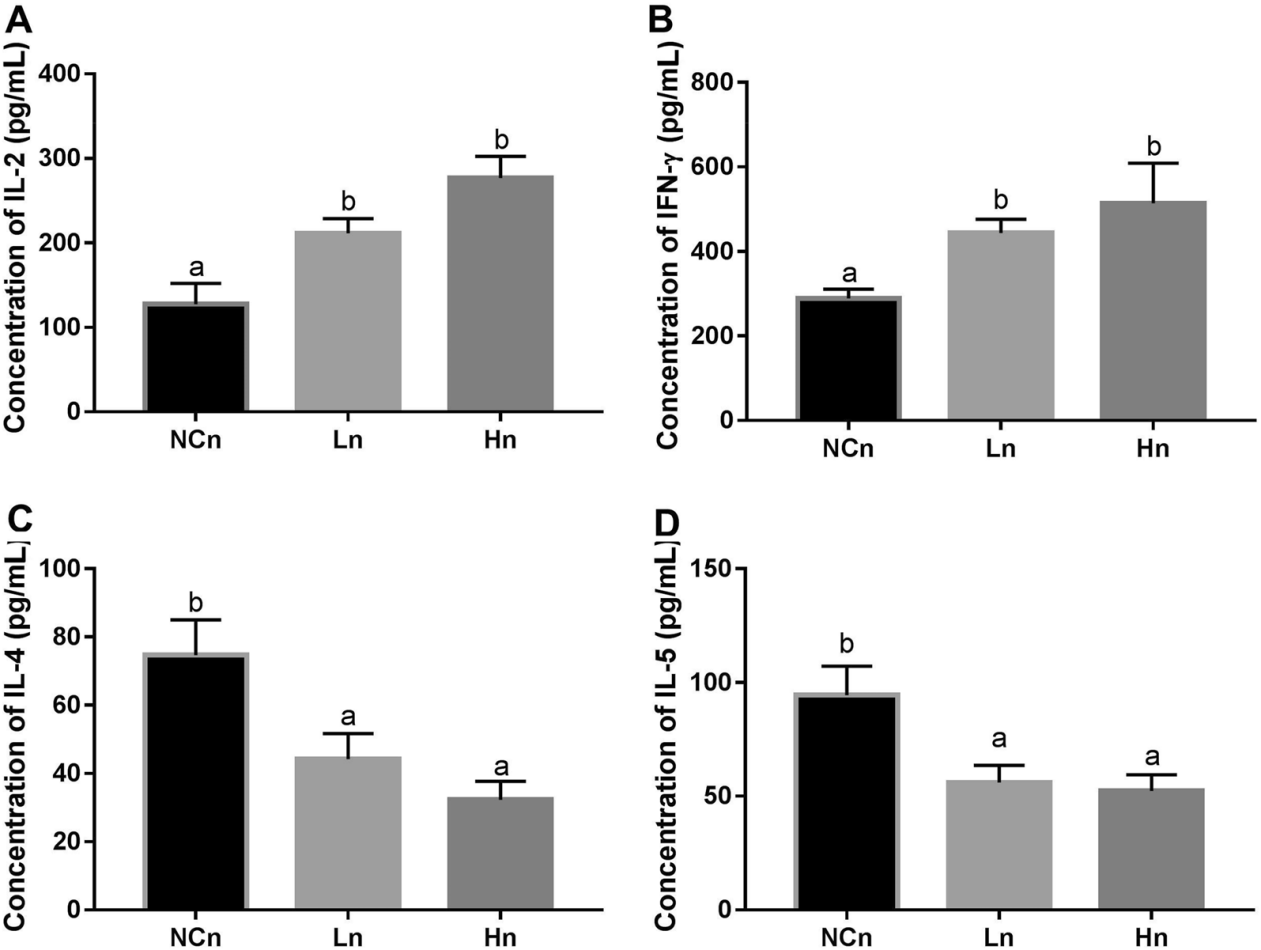
Cytokine profiles of splenocytes from OVA-injected (s.c.) mice with additional intranasal administration ex vivo. Levels of the cytokines (**A**) IL-2, (**B**) IFN-γ, (**C**) IL-4, and (**D**) IL-5 in the supernatant. *NCn* mice gavaged with saline, negative control, *Ln* mice gavaged with low-dose LcSFB, *Hn* mice gavaged with high-dose LcSFB. Different superscript letters (a, b) indicate significant differences at *p* < 0.05; one-way ANOVA with a Tukey HSD post-hoc tests (*n* = 10).

Cytokines produced by Th1 and Th2 cells were determined to further investigate the effect of LcSFB on regulation of Th1 and Th2 cells. First, we confirmed that a Th2 bias was induced in the NCn group, as the increases in the levels of the Th2-related cytokines (IL-4 and IL-5) were much higher than the changes in Th1-related cytokines (IL-2 and IFN-γ) in the NCn group compared to the untreated and NCi mice (Supplementary Table 1). Moreover, LcSFB reversed this trend by increasing Th1 cytokines (Fig. 6A,B) and decreasing Th2 cytokines (Fig. 6C,D) in mice who received the nasal OVA treatment. Interestingly, LcSFB enhanced the levels of Th2 cytokines in the mice that were only injected with OVA (Fig. 3C,D). Therefore, LcSFB has the opposite effects on Th2 cells in OVA-injected mice with and without nasal OVA treatment.

### LcSFB attenuates asthma in OVA-injected mice that also receive nasal OVA treatment

**Figure 7:**
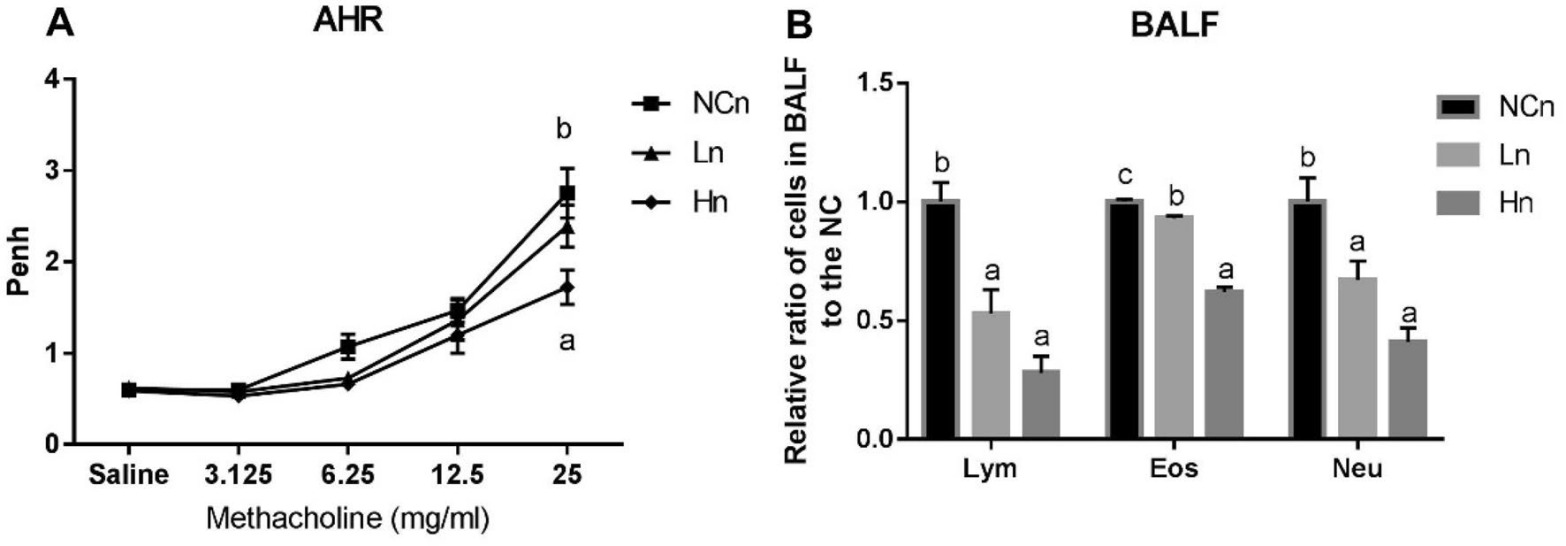
Airway hyperresponsiveness (AHR) (**A**) and immune cells in bronchoalveolar lavage fluid (BALF) (**B**) of OVA-injected (s.c.) mice with additional intranasal administration. *NCn* mice gavaged with saline, negative control, *Ln* mice gavaged with low-dose LcSFB, *Hn* mice gavaged with high-dose LcSFB. Different superscript letters (a, b, c) indicate significant differences at *p* < 0.05; one-way ANOVA with Tukey HSD post-hoc tests (*n* = 10).

LcSFB lowered several biomarkers of asthma, such as high IgE, IgG, and IgM levels and a high Th2/Th1 ratio in OVA-injected mice that received the nasal OVA treatment. Thus, airway hyperresponsiveness (AHR) and bronchoalveolar lavage fluid (BALF) were assessed to further evaluate whether LcSFB improved asthma. An increase in AHR, a hallmark of asthma, was observed in the NCn group compared to untreated mice (Supplementary Fig. 1), which confirmed asthma was successfully induced in the NCn mice. Our results also revealed that mice fed a high dose of LcSFB had significantly lower penh and ratios of lymphocytes, eosinophils, and neutrophils in BALF (Fig. 7A). The ratios of lymphocytes, eosinophils, and neutrophils were also decreased in the low-dose group compared with the NCn group (Fig. 7B). Therefore, LcSFB attenuated the severity of asthma in OVA-injected mice that also received the nasal OVA treatment.

## Discussion

Probiotics have been reported to regulate the immune system^17^. In this study, we compared two antigen presentation models and demonstrated that anti-OVA immunity was activated by both the injection model and injection plus intranasal administration model. However, LcS enhanced the immune responses against OVA in the OVA-injected mice, but attenuated enhancement of the anti-OVA immune responses in the mice both injected with and intranasally administered OVA. These results suggest that the ability of LcS to induce or reduce immunity depends on the antigen presentation route. Thus, it is crucial to consider the route of antigen presentation when probiotics are used to boost immune responses. However, the effects of probiotics on immune responses vary. Probiotics have been shown to extenuate allergies by inducing immune tolerance and a Th1 bias in the Th1/Th2 ratio^18^. However, several studies have demonstrated that probiotics enhance immune responses against pathogens^19^. For example, LcS was shown to prevent the development of asthma and promoted anti-viral immunity^13^. As the same probiotics have been reported to function differently in terms of immunity in various studies^14^, it is important to compare different models in the same study to more precisely understand the mechanisms by which probiotics affect immune regulation.

Immune responses against extracellular antigens are promoted by Th2 cells. Th2 cells secrete cytokines to enhance humoral immune responses to extracellular parasites and bacterial infections^20^. However, Th2 cell hypersensitivity is involved in the allergic reactions that cause asthma. Therefore, prevention of excessive Th2 cell activity against allergens should improve asthma^21^. Treg cells represent appropriate candidates to ameliorate asthma, as these cells are responsible for immune tolerance, including suppression of Th2 cells^22^. In the present study, LcSFB increased Treg cells and decreased Th2-related cytokines in the mice injected with and intranasally administered OVA, which suggests that LcS reduces the activities of Th2 cells via Treg cells. On the other hand, Treg cells were not influenced by LcSFB in the OVA-injected mice that were not intranasally administered OVA. Treg cells play a major role in induction of immune tolerance and have better plasticity in the mucosal immunity compared to systemic immunity^23^. Therefore, antigens at mucosal surfaces typically induce immune tolerance, whereas injected antigens tend to enhance confrontational immune responses^24^. LcS may play an auxiliary role in OVA-induced immune responses. Thus, LcS induced Treg cells and immune tolerance in the mice exposed to OVA via injection plus intranasal administration, but induced anti-OVA immunity and had no influence on Treg cells in the mice administered OVA by injection only. Another possible barrier to these two OVA-administrated routes is that LcS also interacts with the mucosal immune system, so LcS should more strongly induce mucosal Treg cells. Hence, immune tolerance was only observed when mucosal Treg cells were exposed to OVA. Therefore, more studies are needed to understand the mechanisms of action of LcSFB in immune regulation in the future.

A previous study suggested LcS exerts asthma-attenuating effects^14^. Although some parameters of asthma were improved by administration of 1 × 10^9^ heat-killed LcS cells/mouse, the levels of IgG and Th1-related cytokines and the percentage of allergy-related cells in BALF were not significantly different between the control and LcS-treated mice. In our study, live LcS at much lower doses (~ 4.0 × 10^7^ to 1 × 10^8^ colony forming units [CFU]) was given to each mouse. However, we noted significant improvements in several parameters of asthma, including the levels of Th1- and Th2 -related cytokines, IgG, IgE, and IgM levels, and the percentages of allergy-related cells in BALF. Therefore, the ability of live LcS to reduce asthma appears to far exceed that of heat-killed LcS. This result supports the concept that live probiotics are more efficient compared to the reported effects of heat-killed probiotics in previous research^14^. However, further experiments using both live and dead LcS are necessary to confirm this hypothesis.

LcS has also been shown to increase immune responses against the influenza virus. Hori et al*.* (2002) showed that administration of LcS via injection induced innate immune responses that reduced viral titers^13^. Shida et al*.* (2015) also reported that daily intake of fermented milk containing LcS increased the activity of NK cells and levels of IgA, and thus reduced the incidence and duration of upper respiratory tract infections^25^. Our results further demonstrate that supplementation with LcS could enhance adaptive immune responses after OVA injection. Therefore, LcS promoted both innate and adaptive immunity against the injected exogenous antigen. Accordingly, this raises the question of whether the probiotic LcS could boost the efficacy of influenza vaccinations. The answer might be negative, as the route of infection of the influenza virus is via the mucosa; however, our results indicate LcS induced the establishment of immune tolerance in the mice exposed to OVA injection plus intranasal administration. However, the immune responses against viral infection involve a variety of different immune mechanisms. Thus, additional investigations are necessary to assess the potential of LcS for the prevention of influenza. In conclusion, this is the first study to report that distinct antigen presentation models can cause the same probiotic to have opposing effects on immunity. This fact should be taken into account when developing probiotic products for immune regulation.

In conclusion, the effects of LcS were influenced by how the antigen was presented. Our results suggest LcS could be applied to alleviate the symptoms of asthma, but the application of this probiotic as an immune booster needs to be carefully considered.

## Materials and methods

### LcS and experimental animals

**Figure 8:**
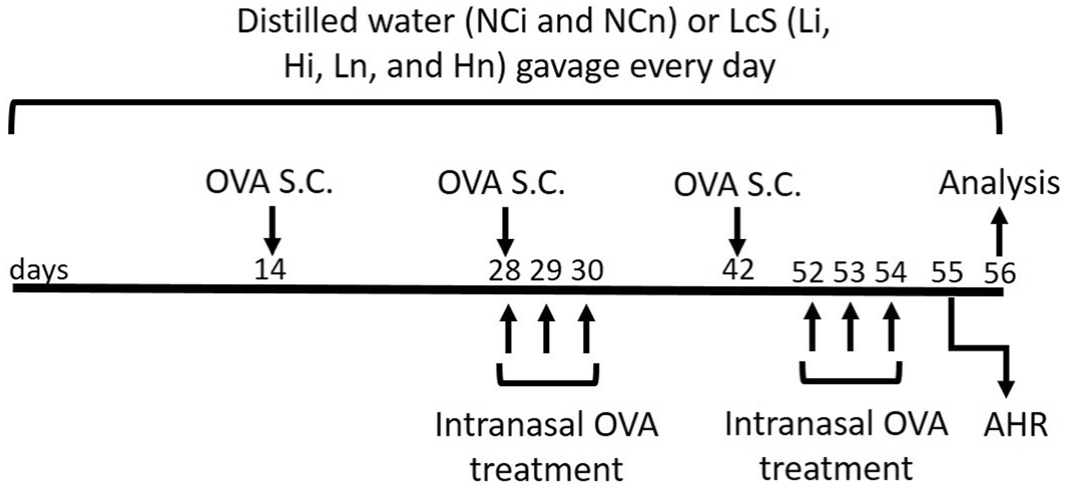
Schematic illustration of the experimental protocol. Briefly, BALB/c mice received either distilled water or *Lactobacillus casei* Shirota fermented beverage (LcSFB) every day. Ovalbumin (OVA) was given to all groups by subcutaneous (s.c.) injection on the 14th, 28th, and 42nd days. Moreover, the NCn, Ln, and Hn groups were treated with OVA via the intranasal route on the 28th, 29th, 30th, 52nd, 53rd, and 54th days. Airway hyperresponsiveness (AHR) was assessed in the NCn, Ln, and Hn groups on the 55th day. The mice were sacrificed on the 56th day, and the proliferation of splenocytes, percentages of regulatory T (Treg) and T helper (Th) lymphocytes in splenocytes, OVA‐specific antibodies, and cytokines were measured.

LcS is present in Yakult 300 light yogurt beverage, which was purchased from Yakult Taiwan (Taipei, Taiwan). The LcS samples were created by freeze-drying 125 mL of Yakult 300 light beverage containing 1.25 × 10^10^ CFU to 11.88 g. The freeze-dried samples were adjusted to 2.44 g (2.5 × 10^9^ CFU) in 1 mL distilled water, and the CFU of LcS was confirmed just before beginning of this study. Eight-week-old BALB/c mice were purchased from LASCO (Ilan, Taiwan) and housed in standard laboratory conditions (12/12-h light/dark cycle, 22–24 °C, 40–60% humidity). The mice were fed a commercially available diet (local supplier), and sterile water was provided ad libitum. Sixty female mice were divided into six groups (namely the NCi, Li, Hi, NCn, Ln, and Hn groups). The mice in each group (*n* = 10) were equally divided into two cages (5 mice/cage) to provide appropriate space for the mice. The groups for the first antigen presentation model (the NCi, Li, and Hi groups) received OVA by injection to simulate injective vaccination. The other antigen presentation model included the NCn, Ln, and Hn groups, which were given OVA by injection plus intranasal administration to imitate the mouse model of allergic asthma. OVA (≥ 98%, Grade V, A5503, Sigma-Aldrich, Saint Louis, MO, USA) was administered via subcutaneous (s.c.) injection at 20, 60, and 60 µg/mouse in the second, fourth, and sixth weeks, respectively. Moreover, each mouse in the NCn, Ln, and Hn groups was additionally treated with 50 µL of 5% OVA per day via intranasal administration for 3 days in the fourth week and 4 days before sacrifice. The OVA treatment protocol was based on our pilot study that successfully induced asthma, as AHR and BALF were significantly higher in the OVA-treated mice than the non-treated mice in the pilot study (Supplementary Table 2). Mice in the NCi and NCn groups were fed 200 µL of distilled water, and mice in the Li and Ln groups were fed a low dose of LcSFB (2.5 × 10^9^ CFU/kg body weight [BW]) of LcSFB sample suspended in 200 µL of distilled water. Moreover, the mice in the Hi and Hn groups were given a high dose of LcSFB (5 × 10^9^ CFU/kg BW of the LcSFB sample dissolved in 200 µL of distilled water). The doses of LcSFB were decided according to the recommend dose of LcS, which is 1.25 × 10^10^ CFU/day/person according to a report by the US Food and Drug Administration^26^. Assuming the average human weight of 60 kg, the dose for a mouse is 1.25 × 10^10^ CFU/person/day × 12.3 (conversion constant between mice and humans) ÷ 60 kg/person = 2.5 × 10^9^ CFU/kg/day. As 2.5 × 10^9^ CFU/kg/day of LcS was a relatively low dose in previous studies exploring the immunoregulatory effects of LcS^14,27,28^, 2.5 × 10^9^ CFU/kg/day and double this dose were selected for this study. The LcSFB sample was given to the mice via gavage for 8 weeks. Blood was collected in the 8^th^ week, and the sacrifice was performed by CO_2_ inhalation at the end of the eighth week. The experimental procedure is shown in Fig. 8. Briefly, BALB/c mice received either distilled water or *Lactobacillus casei* Shirota fermented beverage (LcSFB) every day. Ovalbumin (OVA) was given to all groups by subcutaneous (s.c.) injection on the 14th, 28th, and 42nd days. Moreover, the NCn, Ln, and Hn groups were treated with OVA via the intranasal route on the 28th, 29th, 30th, 52nd, 53rd, and 54th days. Airway hyperresponsiveness (AHR) was assessed in the NCn, Ln, and Hn groups on the 55th day. The mice were sacrificed on the 56th day, and the proliferation of splenocytes, percentages of regulatory T (Treg) and T helper (Th) lymphocytes in splenocytes, OVA‐specific antibodies, and cytokines were measured.

### MTT assay

Splenocytes were isolated from the mice, seeded at 2 × 10^6^ cells/well into 24-well plates, and incubated with 20 μg/mL/well OVA. After 72 h, 100 μL of 5 mg/mL 3-(4,5-dimethylthiazol-2-yl)-2,5-diphenyltetrazolium bromide (MTT, Sigma-Aldrich) was added into each well and incubated at 37 °C for 3.5 h. The cells were added to 400 μL of MTT solvent (4 mM HCl mixed with isopropanol) and shaken at room temperature for 15 min. Absorbance was read at 590 nm with a reference filter of 620 nm using an ELISA reader (PerkinElmer, Waltham, MA, USA).

### Measuring OVA-specific antibodies in serum

The serum levels of OVA-specific antibodies were determined using ELISA kits purchased from Chondrex (Redmond, WA, USA), including an Mouse Anti-OVA IgE Antibody Assay Kit, Mouse Anti-OVA IgA Antibody Assay Kit, Mouse Anti-OVA IgM Antibody Assay Kit, and Mouse Anti-OVA IgG Antibody Assay Kit. Briefly, the 96-well plates were coated with OVA at 4 °C overnight. Then, the plates were washed with PBST solution (phosphate-buffered saline with 0.1% Tween^®^ 20) and blocked with Blocking Buffer for 1 h at 37 °C. After washing, 100 μL aliquots of sera at appropriate dilution ratios were added, and the plates were incubated at 37 °C for 1 h. Next, the plate was washed with PBST and Detection Antibody was added. The plates were incubated at 37 °C for 1 h and then washed. Subsequently, TMB solution was added and incubated for 25 min at room temperature followed by three washes. The reactions were terminated using Stop Solution, and absorbance was read at 450 nm using an ELISA reader (BioTek, Winooski, VT, USA). All samples were run in triplicate.

### Measurement of cytokines

Splenocytes were collected in the eighth week and 2 × 10^6^ cells/well were cultured in the presence of OVA (20 μg/mL/well) for 72 h. IL2, interferon-γ (IFN-γ), IL-4, and IL-5 levels were assayed using ELISA kits (BioLegend) following the manufacturer's protocol. All samples were assessed in triplicate.

### Immune cell detection

Immune cells were detected using antibodies against CD3-FITC/CD4-PE (17A2/RM4-5, BioLegend) (Th cells), and CD3-FITC/CD4-PE/FOXP3-PE-Cy5 (FJK-16s, eBioscience) (Treg cells). To identify Th cells, splenocytes were incubated with different combinations of antibodies for 20 min, and the supernatant was removed via centrifugation at 400*g* for 5 min. After being washed twice with PBST, the cells were re-suspended in 0.5 mL of PBS. Treg cells were stained using the eBioscience™ Foxp3/Transcription Factor Staining Buffer Set (eBioscience), according to the instruction manual. Briefly, cells were stained with CD3-FITC/CD4-PE following the same procedure for staining Th cells. Then, the cells were fixed and permeabilized using IC Fixation Buffer and Permeabilization Buffer, respectively. After being washed twice, the cells were incubated with FOXP3-PE-Cy5 antibody for 30 min. The cells were subsequently washed twice, resuspended, and stained cells were detected utilizing a BD FACSCanto II flow cytometer (BD, Franklin Lakes, NJ, USA). Single stained controls were used to establish fluorescent compensation parameters. Unstained cells were used as negative controls.

### Airway hyperresponsiveness

AHR was assessed in unrestrained mice using whole-body barometric plethysmography (Model PLY 3211; Buxco Electronic Inc., Sharon, CT, USA) that recorded enhanced pause (Penh). Pulmonary resistance was calculated by changing the chamber pressure via administration of methacholine during inspiration and expiration. After a brief rest in the chamber, the mice received an initial baseline administration of saline followed by increasing doses of nebulized methacholine. During the exposure period, each mouse was sequentially given 0, 3.125, 6.250, 12.500, and 25.000 mg/mL of methacholine. Mice remained in the chamber for 3 min, and their respiratory rate was counted. Finally, the Penh values were averaged and reported as percentages of the baseline saline values.

### Bronchoalveolar lavage fluid isolation

BALF was isolated by lavage with 1 mL of PBS. The cells in the BALF were centrifuged (4000*g*, 5 min), transferred onto slides, fixed, and stained using Liu’s stain kit (Shin-Yung medical instruments, Taipei, Taiwan). Standard morphological criteria were used to classify the individual leukocytes.

### Statistics

The data were analyzed using one-way ANOVA with Tukey HSD post-hoc tests using SPSS 22.0 (IBM, Armonk, NY, USA). The data are presented as mean ± standard error of the mean (SEM). A *p* value < 0.05 was considered to be statistically significant; values marked with different superscript letters are significantly different.

### Ethical approval

All of animal experiments were performed by well-trained investigators and in accordance with protocols approved by the Institutional Animal Care and Use Committee (IACUC) of Shih Chien University (IACUC-10509). The authors complied to the ARRIVE guidelines for reporting animal research^29^.

## Supplementary Information

Supplementary Information.
