# Supplementary material for: The immunomodulatory effects of long-term supplementation with Lactobacillus casei Shirota depend on ovalbumin presentation in BALB/c mice
Source: Sci Rep. 2021 Sep 30;11:19478. doi: 10.1038/s41598-021-98791-2 (PMC8484482; doi:10.1038/s41598-021-98791-2)
Supplement: Supplementary file 1 — Supplementary Information. [file 41598_2021_98791_MOESM1_ESM.pdf]

Supplementary table 1. The levels of OVA-induced cytokines in the untreated, NCi, and NCn mice and the fold change.

	Th1 cytokines		Th2 cytokines	
	IL-2 (pg/ml)	INF-γ (pg/ml)	IL-4 (pg/ml)	IL-5 (pg/ml)
Untreated	57.8±22.8	15.0±1.7	2.1±0.3	3.0±0.7
NCi	144.9±18.0	343.3±72.1	43.6±0.4	42.1±5.3
NCn	127.6±24.7	289.0±22.1	74.7±10.3	94.4±12.7
	IL-2	INF-γ	IL-4	IL-5
NCn/Untreated	2.21	19.25	36.11	31.16
NCn/NCi	0.88	0.84	1.71	2.24

Supplementary table 2. The airway hyperresponsiveness (AHR) (A) and immune cells in bronchoalveolar lavage fluid (BALF)

	OVA-treated group	Untreated group
AHR (penh)	2.6±0.3*	1.7±0.2
Lymphocytes (%)	8.5±0.72*	4.2±0.8
Eosinophils (%)	167.5±1.54*	1.6±0.52
Neutrophils (%)	9.0±0.9*	1.5±0.4

* Significantly different from the untreated group, $p<0.05$

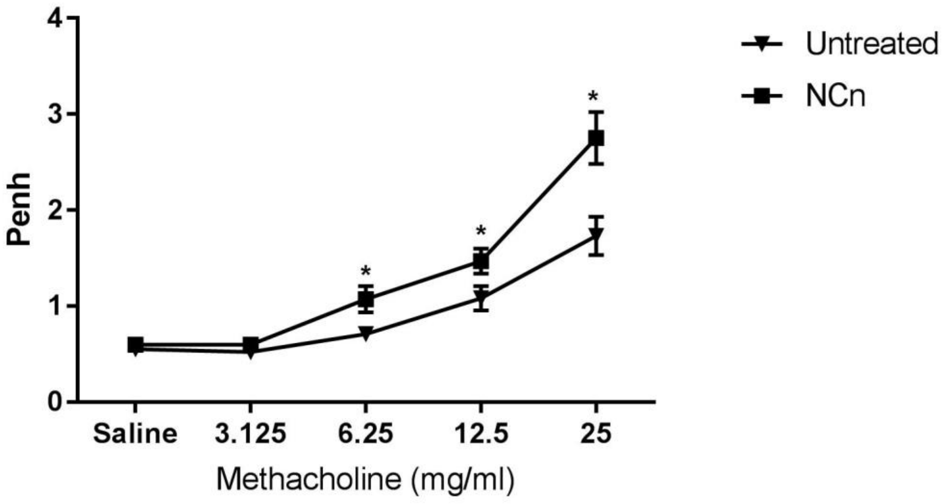

Supplementary figure 1. The AHR of OVA-untreated mice and OVA-injected (s.c.) mice with the additional intranasal challenge. * Significant difference from the untreated mice at $p < 0.05$ according to two-tailed Student's t-test (n = 10).
